# Supplementary figures and images for: Transcriptomic analysis of benznidazole-resistant Trypanosoma cruzi clone reveals nitroreductase I-independent resistance mechanisms
Source: PLoS One. 2025 Feb 18;20(2):e0314189. doi: 10.1371/journal.pone.0314189 (PMC12005674; doi:10.1371/journal.pone.0314189)

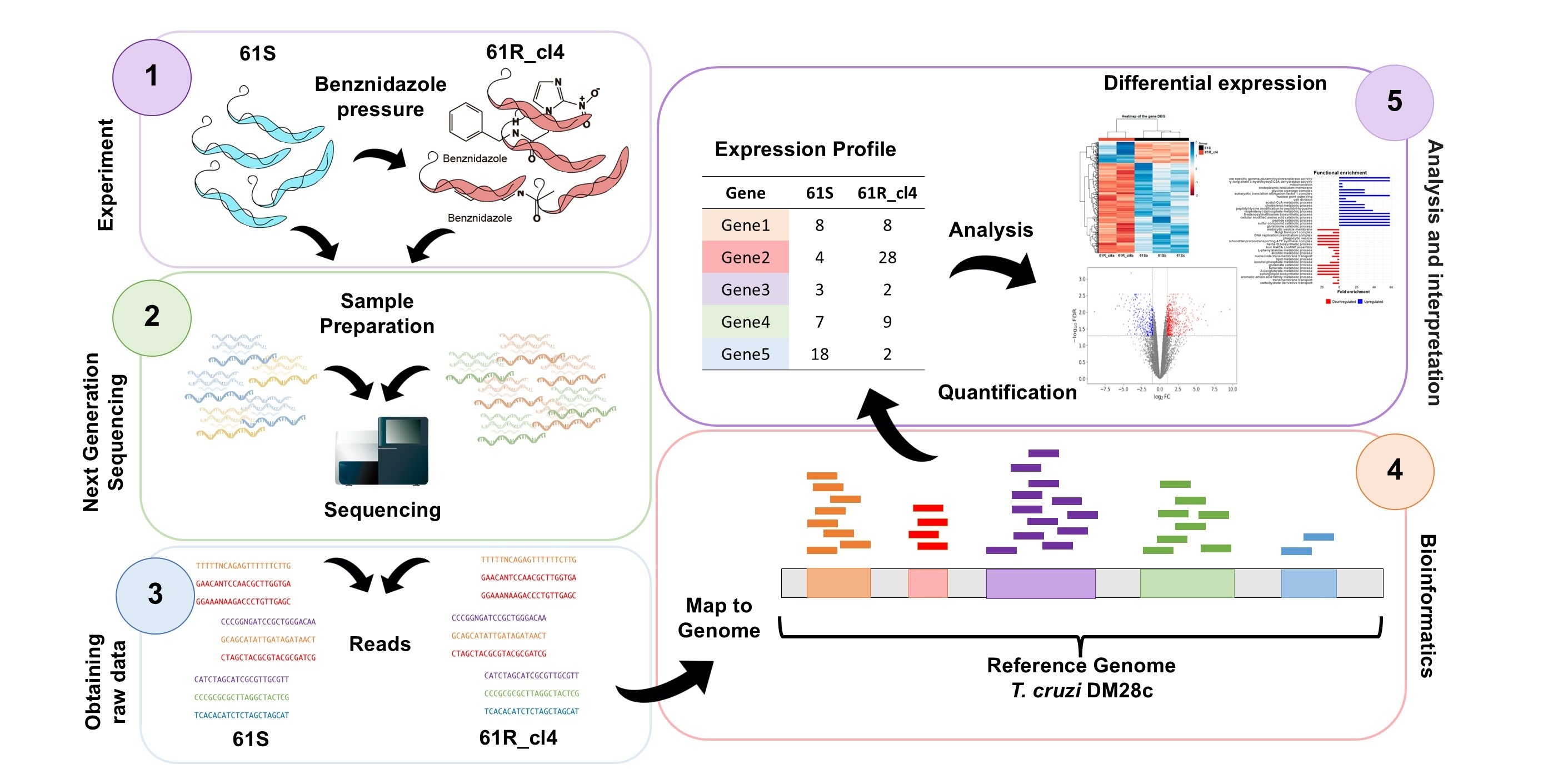

Supplement: S1 Fig — 1. To generate benznidazole resistance from the 61S susceptible clone epimastigotes were subcultured every week under selective pressure until a resistant clone (61R_cl4) was isolated. 2. RNA from both clones was extracted and sequenced using Illumina NovaSeq 6000 platform with paired reads methodology. 3. The quality of the reads obtained was evaluated and the primers, adapters and sequences with a Phred value lower than Q30 were removed. 4. Reads were individually aligned against the T. cruzi Dm28c reference genome (2018) from DTU TcI. 5. Differential expression analysis was performed. (TIF) [file pone.0314189.s001.tif]

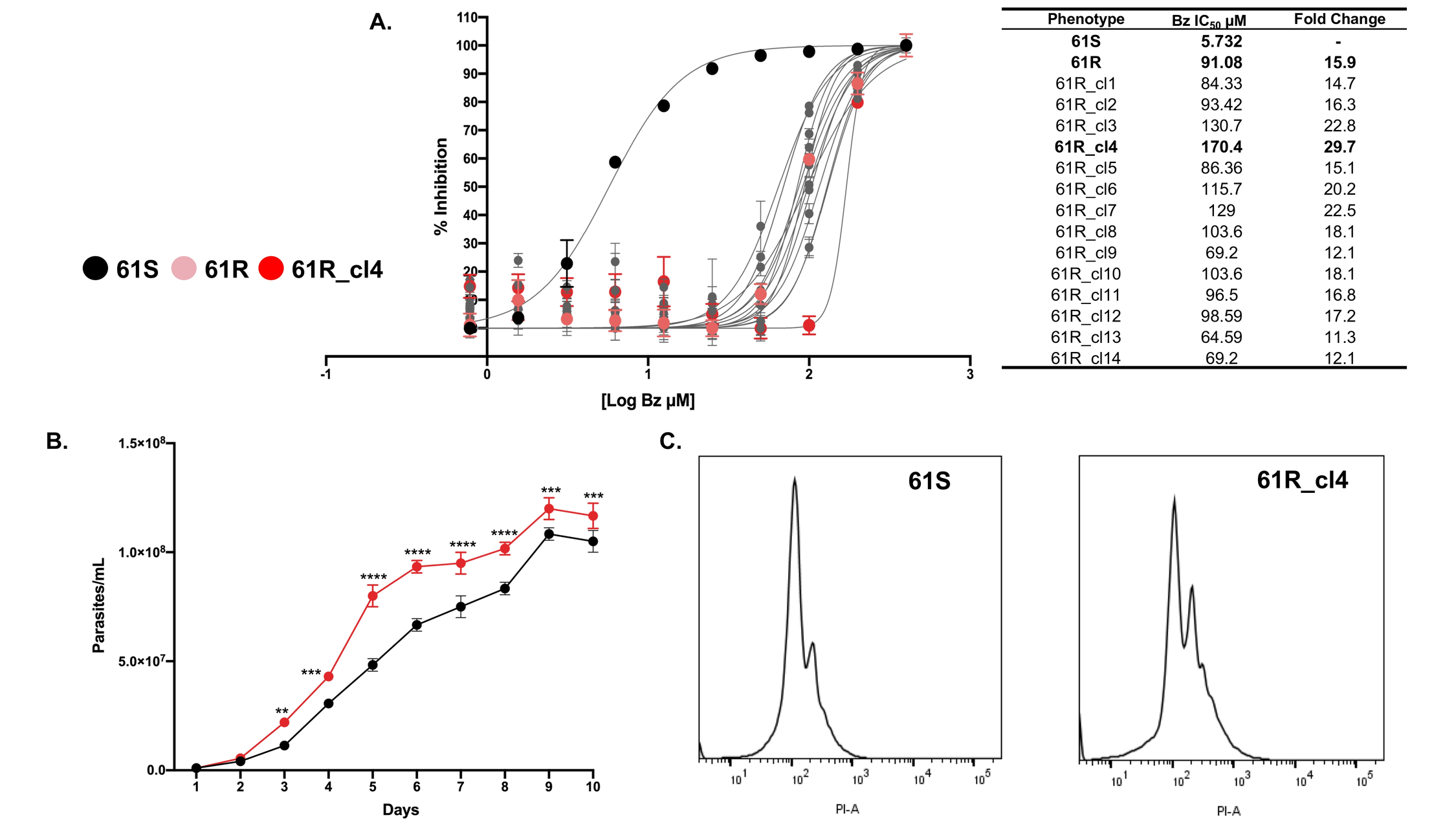

Supplement: S2 Fig — A. The growth inhibition percentage of T. cruzi parasites at Bz was evaluated by alamarBlue and calculated by GraphPad Prism 8.0 from one sensitive clone (61S; black), one resistant phenotype (61R; pink), and 14 resistant clones (gray). In red is highlighted the clone with the lowest inhibition percentage to the drug (61R_cl4; the most resistant). The fold change in the IC50 was calculated with respect to control parasites (61S). B. Epimastigotes proliferation curves were assessed by counting in the Neubauer chamber every 24 hours for ten days. Statistical significance was determined using two-way ANOVA with Sidak´s multiple comparisons test, performed in GraphPad Prism 8.0. ****p < 0.0001; ***p < 0.001; **p < 0.01. Sensitive parasites (61S) are represented in black, and resistant parasites in red (clone: 61R_4). C. Flow cytometry analysis of cell cycle progression. The 61S and 61R_cl4 populations were synchronized with HU for 16 h, and readings were performed using a BD LSRFortessa Cell Analyzer flow cytometer. The graphs showed the median fluorescence intensity of propidium iodide (PI). (TIF) [file pone.0314189.s002.tif]

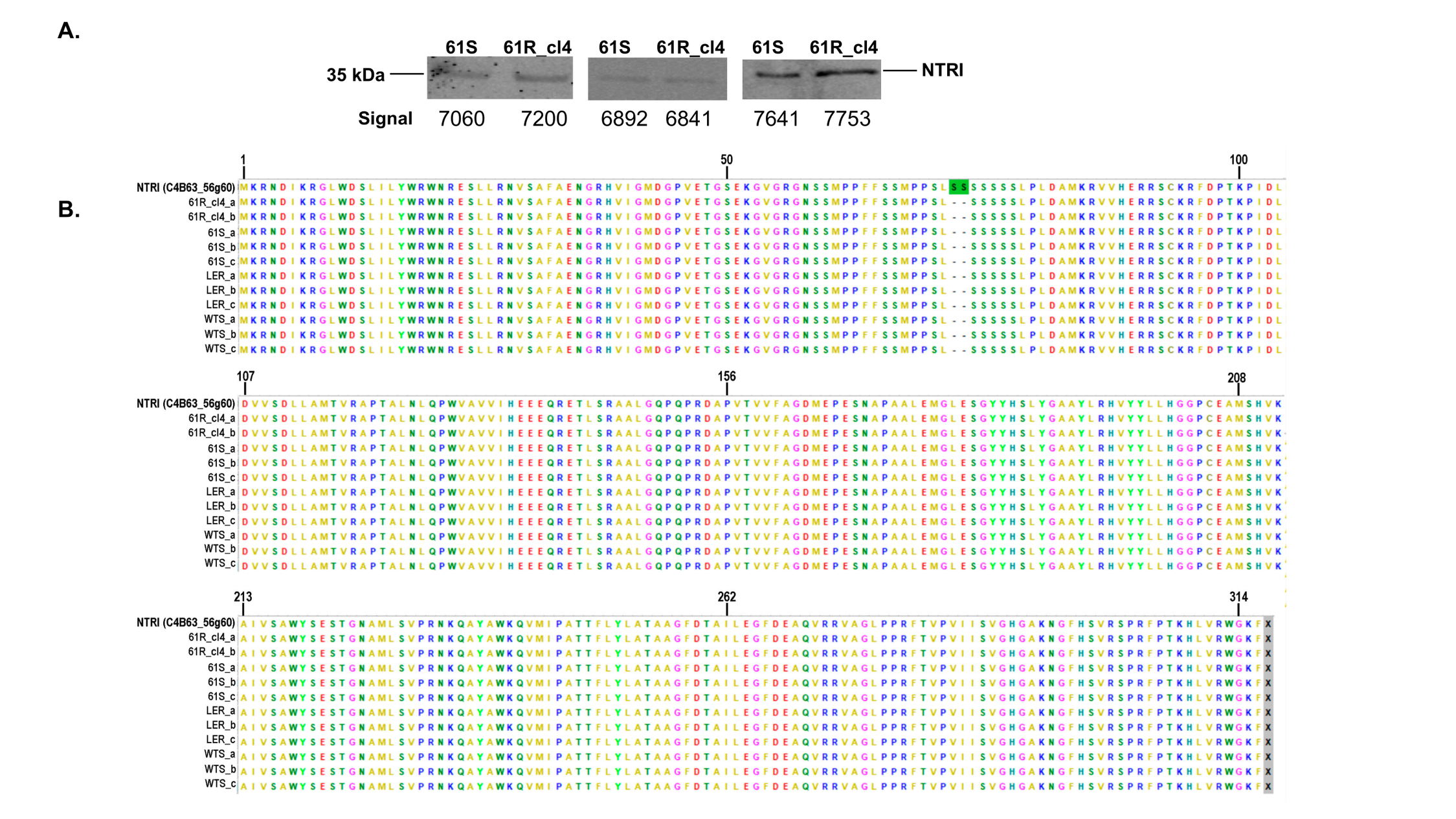

Supplement: S3 Fig — A. Expression level of NTRI gene from sensitive and resistant parasites determined by WB using 100 μg of total proteins from each clone. The intensity of the bands was quantified in the Odyssey Classic Infrared System. B. Aminoacid alignment from NTRI protein deduced from DNA sequencing of different T. cruzi clones. C4B63_56g60: reference sequence; 61S: susceptible clone; 61R_cl4: resistant clone; LER and WTS correspond to T. cruzi resistant and susceptible clones, respectively, obtained by Lima et al., 2023 [26]. (TIF) [file pone.0314189.s003.tif]

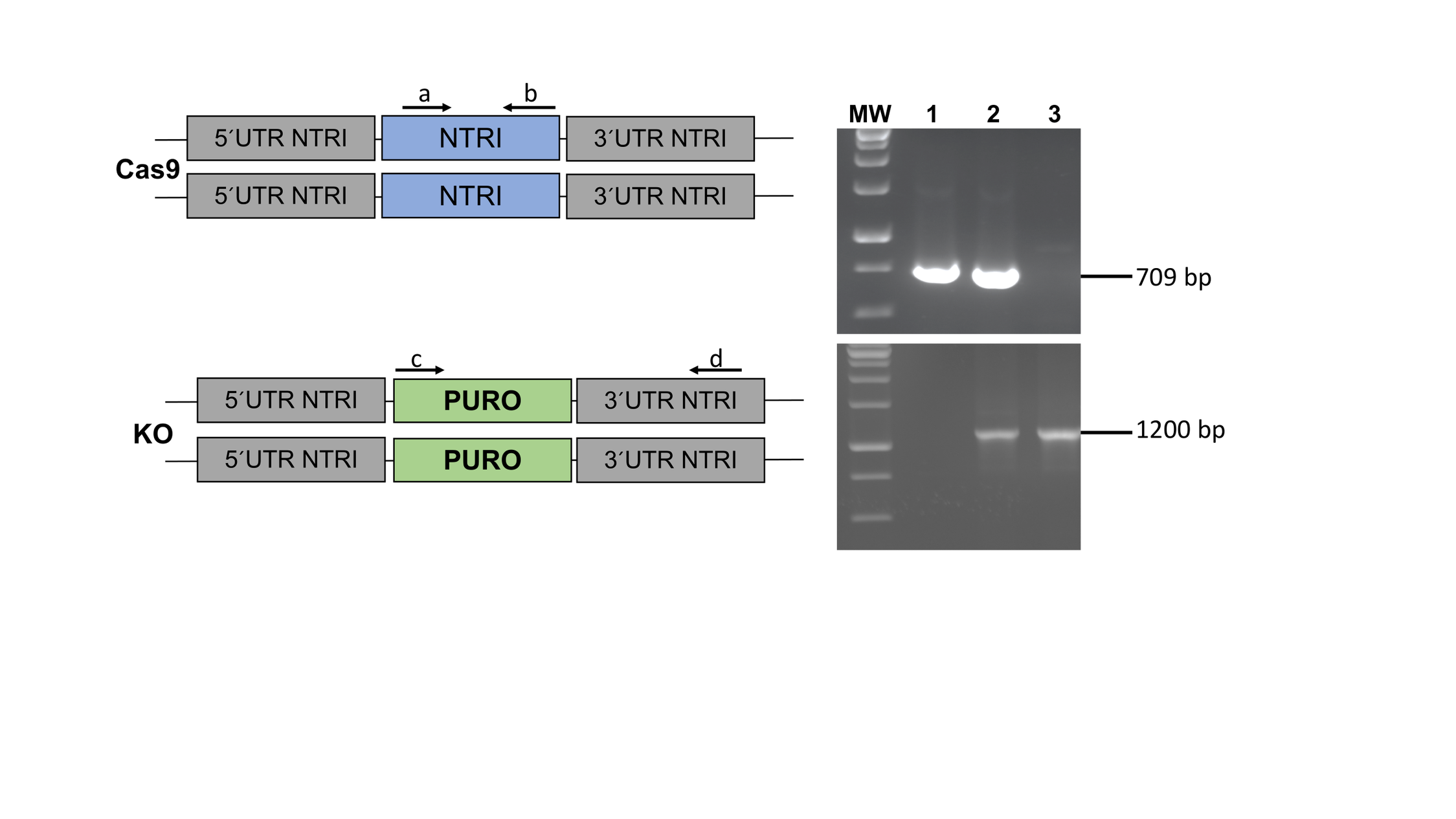

Supplement: S4 Fig — Schematic representations of different PCRs were performed to confirm the knockout of NTRI and the integration of the puromycin (PURO) gene in the NTRI locus. The agarose gel shows the result of the PCR of control parasites (Cas9) (1) and single (2) or double knockout (KO) (3) obtained after the transfection with gRNAs for NTRI gen (above) and puromycin HRTs (below) in the pTREX/Cas9 parasites. All the primer sequences (a, b, c, and d) are listed in the S1 Table . (TIF) [file pone.0314189.s004.tif]

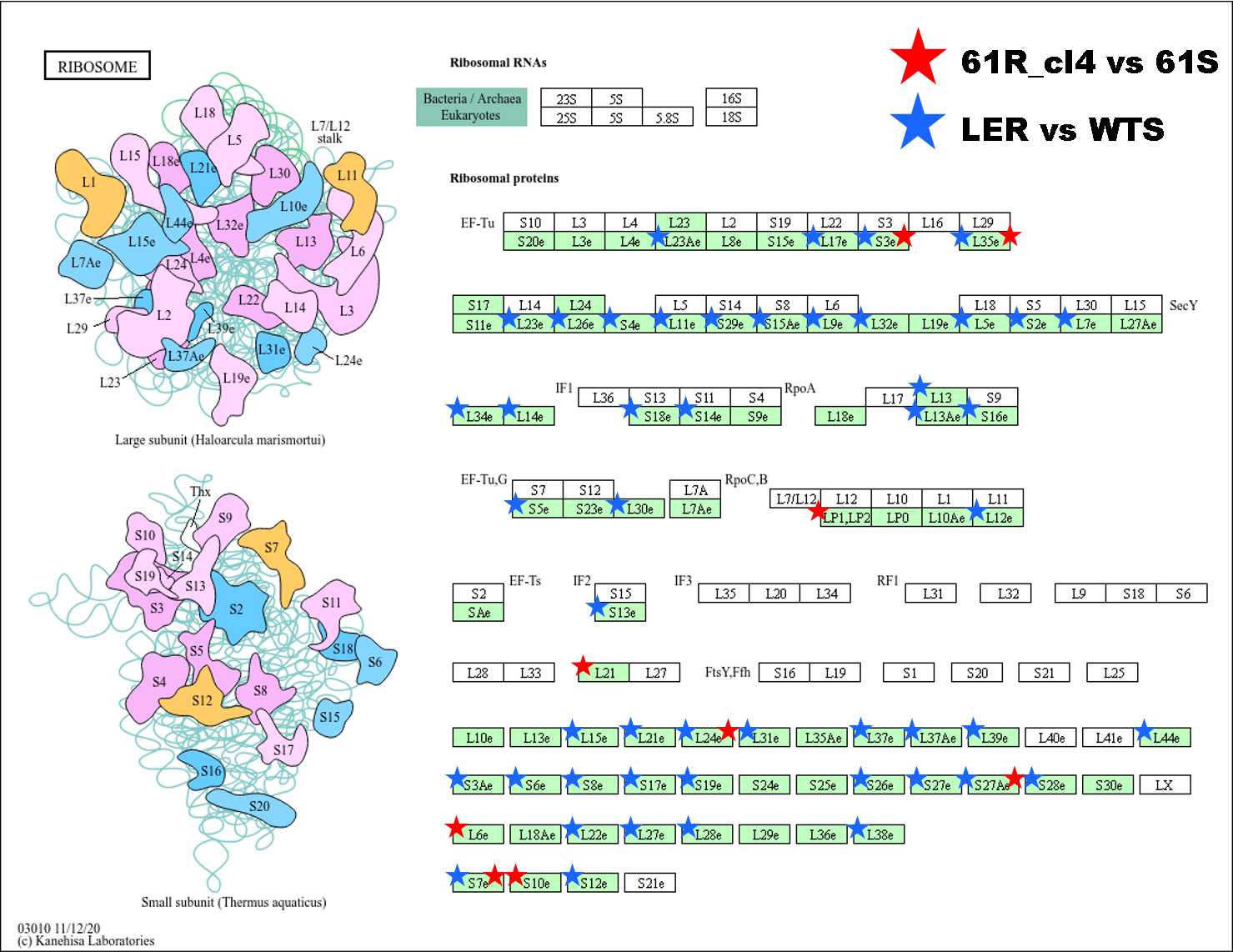

Supplement: S5 Fig — Negatively regulated ribosomal subunits in the 61R_cl4 (red stars) and LER (blue stars) in Trypanosoma cruzi Bz-resistant populations. (TIF) [file pone.0314189.s005.tif]
