# Supplementary material for: Transcriptomic analysis of benznidazole-resistant Trypanosoma cruzi clone reveals nitroreductase I-independent resistance mechanisms
Source: PLoS One. 2025 Feb 18;20(2):e0314189. doi: 10.1371/journal.pone.0314189 (PMC12005674; doi:10.1371/journal.pone.0314189)
Supplement: Attachment — Submitted filename: Response to reviewers.docx [file pone.0314189.s012.docx]

December 29^th^ 2024

**Claudia Patricia Herrera, Ph.D.**

**Academic Editor**

**PLOS ONE**

Dear Editor:

We thank the editor and reviewers for taking the time to review our manuscript PONE-D-24-50847. Their comments have undoubtedly improved the quality of our manuscript. We are glad that they found our manuscript of interest, and our responses to their comments are provided below. In the new version of the manuscript, the changes suggested by the editors and reviewers are indicated in red. Please also note that the order of the Supplementary Figures has changed in this version, as new data have now been included.

Sincerely,

Prof. Ana María Mejía Jaramillo

Universidad de Antioquia

Medellín, Colombia

General changes

Journal Requirements:

When submitting your revision, we need you to address these additional requirements.

1. Please ensure that your manuscript meets PLOS ONE's style requirements, including those for file naming.

**R/ The manuscript meets PLOS ONE´s style requirements.**

2. Thank you for stating in your Funding Statement:

“This work was supported by Universidad de Antioquia UdeA, SGR Grant BPIN 2020000100479.”

Please provide an amended statement that declares *all* the funding or sources of support (whether external or internal to your organization) received during this study, as detailed online in our guide for authors at <http://journals.plos.org/plosone/s/submit-now>.  Please also include the statement “There was no additional external funding received for this study.” in your updated Funding Statement.

Please include your amended Funding Statement within your cover letter. We will change the online submission form on your behalf.

**R/ The information was included in the Funding Statement and cover letter.**

3. When completing the data availability statement of the submission form, you indicated that you will make your data available on acceptance. We strongly recommend all authors decide on a data sharing plan before acceptance, as the process can be lengthy and hold up publication timelines. Please note that, though access restrictions are acceptable now, your entire data will need to be made freely accessible if your manuscript is accepted for publication. This policy applies to all data except where public deposition would breach compliance with the protocol approved by your research ethics board. If you are unable to adhere to our open data policy, please kindly revise your statement to explain your reasoning and we will seek the editor's input on an exemption. Please be assured that, once you have provided your new statement, the assessment of your exemption will not hold up the peer review process.

**R/ I will make the data available.**

4. PLOS requires an ORCID iD for the corresponding author in Editorial Manager on papers submitted after December 6th, 2016.

**R /The ORCID iD was included.**

Additional Editor Comments:

From the perspective of drug resistance, is there a possible correlation between different DTUs or strains of the parasite and their levels of resistance or susceptibility?

**R/ Despite many efforts made by our research team and other researchers, we have not found a clear correlation between drug resistance and the DTUs. We have analyzed the IC_50_ to Bz of many strains from different DTUs, and there is no clear pattern between these two features. The following references showed the mentioned above.**

- Gómez-Palacio, A., Lopera, J., Rojas, W., Bedoya, G., Cantillo-Barraza, O., Marín-Suarez, J., Triana-Chávez, O. & Mejía-Jaramillo, A. (2016). Multilocus analysis indicates that *Trypanosoma cruzi* I genetic substructure associated with sylvatic and domestic cycles is not an attribute conserved throughout Colombia. *Infection, Genetics and Evolution,* *38*, 35–43. <https://doi.org/10.1016/j.meegid.2015.11.026>
- Mejia, A. M., Hall, B. S., Taylor, M. C., Gómez-Palacio, A., Wilkinson, S. R., Triana-Chávez, O., & Kelly, J. M. (2012). Benznidazole-resistance in *Trypanosoma cruzi* is a readily acquired trait that can arise independently in a single population. *The Journal of Infectious Diseases*, *206*(2), 220–228. <https://doi.org/10.1093/infdis/jis331>

- Mejia-Jaramillo, A. M., Fernández, G. J., Montilla, M., Nicholls, R. S., Triana-Chávez, O. (2012). Sensibilidad al benzonidazol de cepas *Trypanosoma cruzi* sugiere la circulación de cepas naturalmente resistentes en Colombia. *Biomédica*, *32*, 196-205.

What are the genotypes of the strains used in this study? Discussing these aspects could help clarify this issue.

**R/ The genotype of this strain I-d was included in line 75. However, we have not discussed this issue further since we only used one strain,** **and this is not a naturally resistant strain which would be relevant to discuss.** **In this sense, many authors have used different strains from distinct DTUs to induce resistance (Lima et al., 2023; Mejia et al., 2008; Murta et al., 2006; Nogueira et al., 2008; Wilkinson et al., 2008), which always results in resistance strains, regardless of the parasite genotype. We believe that the relevant feature in this type of study is how the pressure is done, which will determine the different resistance mechanisms of the parasites, which were discussed in the manuscript.**

- Lima, D. A., Gonçalves, L. O., Reis-Cunha, J. L., Guimarães, P. A. S., Ruiz, J. C., Liarte, D. B., & Murta, S. M. F. (2023). Transcriptomic analysis of benznidazole-resistant and susceptible *Trypanosoma cruzi* populations. *Parasites & Vectors*, *16*(1), 167. https://doi.org/10.1186/s13071-023-05775-4
- Mejia, A. M., Hall, B. S., Taylor, M. C., Gómez-Palacio, A., Wilkinson, S. R., Triana-Chávez, O., & Kelly, J. M. (2012). Benznidazole-resistance in *Trypanosoma cruzi* is a readily acquired trait that can arise independently in a single population. *The Journal of Infectious Diseases*, *206*(2), 220–228. <https://doi.org/10.1093/infdis/jis331>
- Murta, S. M. F., Krieger, M. A., Montenegro, L. R., Campos, F. F. M., Probst, C. M., Ávila, A. R., Muto, N. H., Oliveira, R. C. D., Nunes, L. R., Nirdé, P., Bruna-Romero, O., Goldenberg, S., & Romanha, A. J. (2006). Deletion of copies of the gene encoding old yellow enzyme (TcOYE), a NAD(P)H flavin oxidoreductase, associates with in vitro-induced benznidazole resistance in *Trypanosoma cruzi*. *Molecular and Biochemical Parasitology*, *146*(2), 151–162. https://doi.org/10.1016/j.molbiopara.2005.12.001
- Nogueira, F. B., Krieger, M. A., Nirdé, P., Goldenberg, S., Romanha, A. J., & Murta, S. M. F. (2006). Increased expression of iron-containing superoxide dismutase-A (TcFeSOD-A) enzyme in *Trypanosoma cruzi* population with in vitro-induced resistance to benznidazole. *Acta Tropica*, *100*(1–2), 119–132. https://doi.org/10.1016/j.actatropica.2006.10.004
- Wilkinson, S. R., Taylor, M. C., Horn, D., Kelly, J. M., & Cheeseman, I. (2008). A mechanism for cross-resistance to nifurtimox and benznidazole in trypanosomes. *Proceedings of the National Academy of Sciences*, *105*(13), 5022–5027. https://doi.org/10.1073/pnas.0711014105

Have the researchers observed any variations in parasite susceptibility based on its biological stage? While epimastigotes are commonly utilized for studies, could trypomastigotes (the infective form in mammals) exhibit different responses? Additionally, have the researchers detected any differences at the transcriptomic level?

**R/ Thank you for bringing this matter to our attention. In the past, we analyzed different stages in our resistance clones, and we confirmed that although the IC_50_ values were different between amastigotes, epimastigotes and trypomastigotes, the resistance levels were maintained in other biological stages (González, et al., 2017). In this study, we did not evaluate other forms.**

- González, L., García‐Huertas, P., Triana‐Chávez, O., García, G. A., Murta, S. M. F. & Mejía‐Jaramillo, A. M. (2017). Aldo‐keto reductase and alcohol dehydrogenase contribute to benznidazole natural resistance in *Trypanosoma cruzi*. *Molecular Microbiology*, *106*(5), 704–718. https://doi.org/10.1111/mmi.13830

Line 88. Please clarify what you mean by "same experimental and environmental conditions."

**R/ It means that they were under the same conditions (same temperature, same medium, same number of plate-wells) as parasites treated with the drugs. To avoid confusion, we modified this as: “Untreated parasites were used as growth controls.” (Line 84)**

Line 198. It would enhance the discussion by providing more information about the strains and clones analyzed.

**R/ We only used one strain; from this strain, we selected one clone corresponding to the sensitive clone. After the pressure of this clone with Bz, we obtained a resistant phenotype from which we achieved 14 different clones and selected one to perform the experiments. In our results, we described all the experiments done with these two clones (one sensitive and one resistant).**

Line 462. Please replace "Trypanosoma cruzi" with "T. cruzi."

**R/ We replaced it.**

Figures: Please enhance the quality of the figures.

**R/ All the figures were exported as .tiff with a resolution of 600 dpi**

Reviewers' comments:

Reviewer #1: The article presents a very novel work. It is approached from a transcriptomic approach to elucidate the possible mechanisms of resistance to BZ in populations of T. cruzi resistant by pressure to induced BZ combined with CRISPR Cas technology for genetic knock out (It was lacking to exploit these results a little more in the discussion). However, the discussion of the results needs to be strengthened in general by comparing with more related literature. Although one of the most relevant conclusions of the article is to reaffirm what has been reported by other authors, NTR I independent and multifactorial resistance mechanisms. It remains to further nurture this discussion by trying to present the possible alternative mechanisms that were rightly mentioned in the introduction (lines 44-50).

>What is the reason why the authors performed the work with BZ and not with NFX or both (not bad, but to know their reasons would be good). Why were IC50 determinations made for both, but only selective pressure against BZ? What is the reason for making this decision?

**R/ We thank the reviewer for this comment. We only chose Bz to induce resistance since we had previous works with natural and induced resistance with this drug, so we thought it would be interesting to compare the results obtained here with the previous one. Also, we were interested in comparing our results with those obtained by Lima et al. (2023). Additionally, there is more published information about the treatment with Bz than with Nfx in patients as it is the only treatment available in many countries, so we believe it was more appropriate to analyze this drug. However, we confirmed that our resistance clone had cross-resistance with Nfx, which means that some of the mechanisms are true for both drugs. We think it will be engaging in the future to also use Nfx.**

In the methodology it is mentioned that 10 concentrations of Bz and NFX were used, specify which were those concentrations. It also does not specify the presentation in which NFX and BZ were used, pure, tablet or purified from a tablet?

**R/ Thank you for your feedback. We included this information in the manuscript as follows: “In each assay, 10 concentrations of the drugs (0.78-400 μM for Bz or 0.195-100 μM for Nfx) purified from tablets and prepared in DMSO, were evaluated. Each concentration was assessed four times in 96-well plates.” (Line 82).**

>What is the justification for performing the determination of the IC50 at 72 h?

**R/ The IC_50_ (half-maximal inhibitory concentration) is evaluated at 72 hours because it allows cells to divide 1–2 times and observe drug effects without reaching confluence and it will depend mainly on the parasite stage evaluated. In case of no replicative forms it is common to evaluate the IC_50_ at 24 hours.**

>Specify in the methodology what was the concentration of rezarzurin used since only 20 ul/well is mentioned, but this is very ambiguous.

**R/ Thank you for your feedback. We changed this in the manuscript as follows: “Plates were incubated for 72 h at 28 °C, and the effect of the compounds was determined by adding 20 μl/well of alamarBlue (Resazurin sodium salt, catalog number R7017, Sigma Aldrich, St. Louis, USA), prepared in PBS at 0.125 mg/mL and incubating overnight at 28 °C, as was described previously [19].” (Lines 84-87).**

>How do they justify the use of the Epimastigote form and not the trypomastigote form?

**R/ In our experience, we have noticed that the most significant changes related to this type of induced resistance are fixed in the DNA as deletions, mutations, and amplifications, which will change the gene expression in these parasites. In both stages, it is possible to find genes that modify the expression due to resistance. However, since epimastigotes are the replicative form, it will allow us to see more relevant genes involved in this and other process and also it was possible to compared our results with the ones obtained by Lima et al. (2023).**

>While the focus of the article is not to relate this to intra and inter DTUs genetic variability of T. cruzi (suggested to mention this in the introduction as a possible factor that may influence heterogeneity of response to Bz), how scalable or representable are these results for the other DTU's? Would one expect a big change using another DTU or another TcI isolate?

**R/ As mentioned before, despite many efforts made by our research team and other researchers, we have not found a clear correlation between drug resistance and the DTUs. We have analyzed the IC_50_ to Bz of many strains from different DTUs, and there is no clear pattern between these two features. Furthermore, many authors have used different strains from distinct DTUs to induce resistance, which always results in resistance strains independent of the genotype of the parasites. We believe the relevant feature in this type of study is how the pressure is done, which determines the different resistance mechanisms of the parasites, which was discussed in the manuscript. Please, find below some references that showed the mentioned above.**

- Gómez-Palacio, A., Lopera, J., Rojas, W., Bedoya, G., Cantillo-Barraza, O., Marín-Suarez, J., Triana-Chávez, O. & Mejía-Jaramillo, A. (2016). Multilocus analysis indicates that *Trypanosoma cruzi* I genetic substructure associated with sylvatic and domestic cycles is not an attribute conserved throughout Colombia. *Infection, Genetics and Evolution,* *38*, 35–43. <https://doi.org/10.1016/j.meegid.2015.11.026>

- Lima, D. A., Gonçalves, L. O., Reis-Cunha, J. L., Guimarães, P. A. S., Ruiz, J. C., Liarte, D. B., & Murta, S. M. F. (2023). Transcriptomic analysis of benznidazole-resistant and susceptible *Trypanosoma cruzi* populations. *Parasites & Vectors*, *16*(1), 167. https://doi.org/10.1186/s13071-023-05775-4

- Mejia, A. M., Hall, B. S., Taylor, M. C., Gómez-Palacio, A., Wilkinson, S. R., Triana-Chávez, O., & Kelly, J. M. (2012). Benznidazole-resistance in *Trypanosoma cruzi* is a readily acquired trait that can arise independently in a single population. *The Journal of Infectious Diseases*, *206*(2), 220–228. <https://doi.org/10.1093/infdis/jis331>

- Mejia-Jaramillo, A. M., Fernández, G. J., Montilla, M., Nicholls, R. S., Triana-Chávez, O. (2012). Sensibilidad al benzonidazol de cepas *Trypanosoma cruzi* sugiere la circulación de cepas naturalmente resistentes en Colombia. *Biomédica*, *32*, 196-205.

- Murta, S. M. F., Krieger, M. A., Montenegro, L. R., Campos, F. F. M., Probst, C. M., Ávila, A. R., Muto, N. H., Oliveira, R. C. D., Nunes, L. R., Nirdé, P., Bruna-Romero, O., Goldenberg, S., & Romanha, A. J. (2006). Deletion of copies of the gene encoding old yellow enzyme (TcOYE), a NAD(P)H flavin oxidoreductase, associates with in vitro-induced benznidazole resistance in *Trypanosoma cruzi*. *Molecular and Biochemical Parasitology*, *146*(2), 151–162. https://doi.org/10.1016/j.molbiopara.2005.12.001

- Nogueira, F. B., Krieger, M. A., Nirdé, P., Goldenberg, S., Romanha, A. J., & Murta, S. M. F. (2006). Increased expression of iron-containing superoxide dismutase-A (TcFeSOD-A) enzyme in *Trypanosoma cruzi* population with in vitro-induced resistance to benznidazole. *Acta Tropica*, *100*(1–2), 119–132. https://doi.org/10.1016/j.actatropica.2006.10.004

- Wilkinson, S. R., Taylor, M. C., Horn, D., Kelly, J. M., & Cheeseman, I. (2008). A mechanism for cross-resistance to nifurtimox and benznidazole in trypanosomes. *Proceedings of the National Academy of Sciences*, *105*(13), 5022–5027. https://doi.org/10.1073/pnas.0711014105

Reviewer #2: This is the revision of the study entitled 'Transcriptomic analysis of benznidazole-resistant Trypanosoma cruzi clone reveals nitroreductase I-independent resistance mechanisms' by Mejia-Jaramillo et al. The study is robust and well-executed, with the generation of benznidazole-resistant T. cruzi aligning with findings reported in the literature. While the study has some flaws, its findings are valuable to the scientific community as they highlight resistance mechanisms entirely independent of nitroreductase I.

The introduction includes a few ambiguous statements that require clarification. Additionally, the Materials and Methods section lacks crucial details necessary for reproducibility. The Results section also needs refinement to improve the clarity and presentation of the data.

Introduction

Lines 38-43: The end of this paragraph is highly inaccurate. Both Benznidazole and Nifurtimox have satisfactory results in the acute phase of Chagas disease (50-80% efficacy) but debatable efficiency in the chronic phase. The cited percentage of people discontinuing treatment is also unknown to me, as the last published finalized clinical trial BENEFIT (10.1056/NEJMoa1507574) showed a number of 13.4% of treatment discontinuation due to adverse events. If a study showed 20% of treatment discontinuation, please cite it in the text. I would cite clinical trials aiming to test both drugs in a prolonged treatment scheme too (10.1136/bmjopen-2021-052897).
I suggest updating the references and citing more relevant bibliography.
Here are some suggestions:
10.1111/bcp.14700
10.1056/NEJMct1014204
10.3390/vaccines12080870

**R/ We appreciate your comments. The information used in this paragraph is supported by the literature review we did, which was cited in the manuscript. You mentioned that Bz and Nfx have satisfactory results in the acute phase but only with 50-80% efficacy. We are very concerned about whether these percentages are really satisfactory. However, in order to clarify the information, we have decided to improve this paragraph by including the references you recommended, which are more recent, as well others that are more relevant to support the text. We changed the manuscript as: “Both drugs have unsatisfactory results in chemotherapy, being effective in the acute phase but with a cure rate of only 60-85% [1,2]. Unfortunately, these drugs have several drawbacks, such as treatment failure due to resistance, limited efficacy in the chronic stage of the disease, lack of consistency of treatment response in different geographical areas, side effects that cause 15-20% of people to discontinue treatment, and finally the need for prolonged treatment to increase efficacy [3–9].” (Lines 37-43).**

Lines 45-47: “In resistance to Bz and Nfx, the enzyme nitroreductase I (NTRI) has been implicated as the primary gene responsible for resistance in vitro [4,5] and in patients with treatment failure”. The sentence is confusing; I would rephrase it to make it understandable that it is the gene encoding the enzyme nitroreductase that is associated with drug resistance.

**R/ We are very grateful for the reviewer’s recommendation. We modified this sentence to: “In resistance to Bz and Nfx, the gene encoding the enzyme nitroreductase I (NTRI) has been implicated as the primary response for resistance *in vitro* [10,11] and in patients with treatment failure [12].” (Lines 45-47).**

Materials and Methods

Please revise the text to provide catalog numbers, the manufacturer of the kits/reagents used, and complete names and models of the equipment used.

**R/ We included the missing information in the new version.**

The methods section is quite detailed and dense, which may make it challenging for readers to follow. To enhance clarity and accessibility, I recommend including a visual graphic that summarizes the key steps and analytical processes described in the paper. This could help readers better understand the workflow and overall approach at a glance.

**R/ We accepted the reviewer’s recommendation and included a new figure summarizing the key steps (see Fig S1 and lines 688-695).**

Lines 74-81: In this section, it is not clear to me if you added Bz, G418, and Puromycin in the same media or if they were added into different media comprising different groups (one was treated only with Bz, one only with G418 and one only with Puromycin).

**R/ We are very grateful for this comment. However, we want to clarify that, we provided a general description of the drugs used in all the experiments. Then, in each method section, we mentioned the drugs used and the specific procedure performed. To avoid confusion, we removed this information from the general description and only mentioned the drugs in each experiment. Also, we clarified this information in methods as follow: “Two different transfections were performed: the first to overexpress the Cas9 enzyme in the 61S clone using the pTREX/n-Cas9 plasmid [21], selecting parasites with G418 disulfate salt (Catalog number G8168, Sigma Aldrich, St. Louis, USA), and following the conditions described by Lander et al., (2015) [21]. The second transfection was performed with parasites expressing the Cas9 enzyme (61S_Cas9), using specific sgRNA and a homology repair template (HRT) containing the puromycin gene as described below. These parasites were selected with 250 μg/mL G418, and 60 μg/mL puromycin dihydrochloride from *Streptomyces alboniger* (Catalog number P9620, Sigma Aldrich, St. Louis, USA) according to the protocols described by Queffeulou et al., 2024 [22].” (Lines 126-135).**

Please clarify and explain why the use G418 and Puromycin (positive/negative controls? Or selection of a specific genotype?) If they’re for different experiments, please include the methodology for each experiment separately.

**R/ The use of these drugs was to select the positive transfected parasites with the different plasmids, or repair cassettes. Thus, G418 was used to select the parasites transfected with the pTREX-Cas9 plasmid. In the same way, we used puromycin to select knock-out parasites transfected with the repair cassette containing the puromycin gene. We tried to clarify the methodology as we mentioned above, and we also added some references supporting these procedures (Lines 126-135).**

Are M-RATTUS, CO, 91, GAL-80 and 61.SUC different T. cruzi genotypes or clones? Please specify in the text, it is not clear. In that case, was the IC50 for Bz exactly the same value for all strains?

**R/ We only used one wild-type *Trypanosoma cruzi* strain. The full name of this strain following international recommendations, is M-RATTUS/CO/91/GAL-61.SUC., which includes the M. from mammalian, the species from which it was isolated (*R. rattus*), the country (Colombia), the year (1991), the strain name (Gal-61), and the region (Sucre). We mentioned this in line 75. In the rest of the manuscript, we used the short name of one clone isolated from this strain name 61S for easy reading. We obtained the 61R_cl4 clone from 61S as described in lines 98-100.**

Lines 90-91: Cite a reference for the alamarBlue method for the determination of IC50.

**R/ We included this reference in the new version (Reference 19, line 87).**

Lines 101-102: From my understanding, only the 61.SUC clone was selected for Benznidazole pressure experiments, but that is not clear in the text. Please clarify how that decision was made and what led you to that decision.

**R/ We accepted this recommendation and clarified the *T. cruzi* strains and the drug concentrations used for the selection. We used the *T. cruzi* 61S clone because it is sensitive to Bz. We clarified the IC_50_ and fold change for all the clones analyzed (see Figs 1 and S2A) and the decision to select this clone (Lines 200-201).**

Line 152: The numbers do not need to be written; they can be used as numerical numbers.

**R/ We accepted this recommendation.**

Lines 161: Include the name of the kit used for library preparation.

**R/ The library was also prepared for Novogene Company, USA., using the polyA enrichment library method. We clarify this in lines 172-173.**

Results

Overall, if it is possible, I would change the colors to red for up-regulated and blue for down-regulated genes. This is what we most see in omics papers, and I believe it would make it easier for understanding.

**R/ Thanks for this suggestion. However, we do not accept this recommendation because one of our main aims in this paper was to compare with the results presented by Lima et al., 2023. In this paper, they use the same colors as we do. So, we believed it would be very confusing changing the colors.**

Line 187: It’s ‘passages’ instead of ‘passes’.

**R/ We changed this word in the new version.**

Lines 187-198: Please mention the IC50 for each of the groups, corroborating fold-change resistance to Bz. If you mentioned it for Bz, mention the values for Nfx as well.

R/ **We accepted this recommendation and included this information in figure 1 as well in Figure S2.**

The 61S_Cas9, SKO_NTR1, and DKO_NTRI groups were not mentioned in the results, only in the figure legend. You either exclude this data from the graph or present the results in your text.

**R/ Thank you for your feedback. Indeed, we included this information in the methodology and as well in the result. For more clarity, we mentioned the names of knock-out parasites. The 61S_Cas9 corresponds to the control of KO parasites, and we used this as a control in these procedures.**

Lines 223-229: I see that the Figure 1 61S_Cas9, SKO_NTR1, and DKO_NTRI groups are mentioned here. However, I suggest separating the graphs and making another figure (or maybe Figure 1C) to fit this part of the results.

**R/ We agree with this recommendation and changed Figure 1.**

Line 240: Why was the Log2FC chosen of 0.58, when usually the Log2FC chosen for genes is 1?

**R/ In our case, the genes were considered differentially regulated when Log2FC was ≥0.58 (1.5-fold). This parameter has been used previously in other papers, including the one by Lima et al., 2023. We referenced one of these papers in the methods section (reference 23).**

Line 252: What is the importance of showing that the DRGs are annotated as conserved
hypothetical protein (CHP) and unspecified product (UnP)?

**R/ The annotated genes grouped as hypothetical proteins correspond to genes coding for conserved proteins in Trypanosomatids, while that unspecified product corresponds to proteins present only in *Trypanosoma cruzi.* For that reason, we decided to maintain this classification.**

Figure 2C: Please increase the font size for the gene names, as they will be unreadable in the paper.

**R/ We changed this information in the new version.**

Figure 4. I recommend separating each functional group in (A) Transmembrane Transport, (B) Ribosome and (C) Nucleobase metabolic process, and mentioning it separately in the text.

**R/ We are very grateful and accepted this recommendation.**

S4 Fig. Legend description and presentation of results need to be improved. What does the blue, pink and yellow colors in the subunits mean? What question you plan to answer with this analysis?

**R/ The colors blue, pink, and yellow are defined by defect when we used the KEEG analysis. They show the different proteins conforming the ribosome. However, it does not have a relevant information. The significant information is shown on the right side of this figure.**

Figure 6. Please increase the font size for readers.

**R/ We changed this information in the new version.**

Reviewer #3: The manuscript by Mejia-Jaramillo et al. describes the results of an exhaustive study centered in the characterization by RNA-seq analysis to identify genes other than nitroreductase-I (NTRI) involved in benznidazole and nifurtimox activity against Trypanosoma cruzi, the causative agent of American Trypanosomiasis (Chagas disease). The authors conclude that besides intrinsic NTRI toxicity, upregulation of heat shock proteins (HSPs) and of CDC45 in resistant clones could favor more efficient detoxification, DNA replication and repair under Bz/Nfx genotoxic stress. These are novel and very relevant findings in the characterization of molecular mechanisms involved in the resistance to the only two drugs currently available for the etiological treatment of Chagas disease, in terms of the potential prevention of such resistance via combination treatments.

**R/ We are very grateful for the comments on our manuscript.**
