# Supplementary material for: Transcriptomic analysis of benznidazole-resistant Trypanosoma cruzi clone reveals nitroreductase I-independent resistance mechanisms
Source: PLoS One. 2025 Feb 18;20(2):e0314189. doi: 10.1371/journal.pone.0314189 (PMC12005674; doi:10.1371/journal.pone.0314189)
Supplement: Attachment — Submitted filename: Response to reviewers2.docx [file pone.0314189.s013.docx]

January 20^th^ 2025

**Claudia Patricia Herrera, Ph.D.**

**Academic Editor**

**PLOS ONE**

Dear Editor:

We thank the editor and reviewers for taking the time to review our manuscript PONE-D-24-50847. Their comments have undoubtedly improved the quality of our manuscript. We are glad that they found our manuscript of interest, and our responses to their comments are provided below. In the new version of the manuscript, the changes suggested by the editors and reviewers are indicated in red.

Sincerely,

Prof. Ana María Mejía Jaramillo

Universidad de Antioquia

Medellín, Colombia

General changes

Journal Requirements:

Please review your reference list to ensure that it is complete and correct. If you have cited papers that have been retracted, please include the rationale for doing so in the manuscript text, or remove these references and replace them with relevant current references. Any changes to the reference list should be mentioned in the rebuttal letter that accompanies your revised manuscript. If you need to cite a retracted article, indicate the article’s retracted status in the References list and also include a citation and full reference for the retraction notice.

**R/ The references where revised.**

Additional Editor Comments:

Thank you for submitting the revision and addressing the reviewers' comments. I believe the paper is ready for publication; however, I would appreciate it if you could take the last comment from Reviewer 2 into account before final acceptance.

**R/ Thank you. The two comments where addressed. You will find the answer below.**

Reviewers' comments:

Reviewer #1: I have read the revised version, and it is much better and more scientifically robust, I would just like to point out two things to the authors.

1. in relation to resarsurin, the authors state the use of 20 uL of the above mentioned reagent but they still omit the concentration they have of the compound in those 20 uL, please specify.

**R/ Thank you for your feedback. In the last version, we did include the concentration which is 0.125 mg/mL as follows: “Plates were incubated for 72 h at 28 °C, and the effect of the compounds was determined by adding 20 μl/well of alamarBlue (Resazurin sodium salt, catalog number R7017, Sigma Aldrich, St. Louis, USA), prepared in PBS at 0.125 mg/mL and incubating overnight at 28 °C, as was described previously [19].” (Lines 84-87).**

2. Has recently been published the work; Ochoa-Martínez P, López-Monteon A, López-Domínguez J, Manning-Cela RG, Ramos-Ligonio A. Expression Analysis of Thirteen Genes in Response to Nifurtimox and Benznidazole in Mexican Isolates of Trypanosoma cruzi by Digital PCR. Acta Parasitol. 2025 Jan 7;70(1):15. doi: 10.1007/s11686-024-00986-w. Where a review of the activation of various genes in response to stress exerted by BZN and NFX is made, what can the authors discuss their results in relation to this work?

**R/ Thank you for bringing this matter to our attention. We included the reference in the paper and discussed as follow: “However, a recent study using Mexican *T. cruzi* isolates with different Bz and NFx resistance profiles showed by digital PCR that resistant parasites had increased NTRI expression. This was counterbalanced by overexpression of detoxifying enzymes such as SOD and GTS or transporters such as MDR [44]. (Lines 507-511).**

44.	Ochoa-Martínez P, López-Monteon A, López-Domínguez J, Manning-Cela RG, Ramos-Ligonio A. Expression analysis of thirteen genes in response to nifurtimox and benznidazole in Mexican isolates of *Trypanosoma cruzi* by digital PCR. Acta Parasit. 2025;70: 1–11. doi:10.1007/s11686-024-00986-w

Reviewer #2: The author has thoroughly addressed all the suggestions and recommendations provided. Upon further review, I am confident that this article is now suitable for publication in PLOS ONE.

**R/ We are very grateful for the comments on our manuscript.**
